# Detection of *Candida* spp. from peritoneal swabs indicate worse outcome in patients with perforated peptic ulcer: revisiting a longstanding debate

**DOI:** 10.1186/s12893-026-03518-7

**Published:** 2026-01-24

**Authors:** Faruk Koca, Svenja Sliwinski, Konstantin Uttinger, Ekaterina Petrova, Patrizia Malkomes, Michael Hogardt, Volkhard A. J. Kempf, Tamás Benkö, Armin Wiegering, Niels Matthes

**Affiliations:** 1 Department of General, Visceral, Transplant and Thoracic Surgery, Goethe University Frankfurt, University Hospital, Theodor-Stern-Kai 7, Frankfurt am Main, 60590 Germany; 2 Department of Surgery, Ruhr-University Bochum, Knappschaft Kliniken University Hospital Bochum, Bochum, Germany; 3 Institute for Medical Microbiology and Infection Control, Goethe University Frankfurt, University Hospital, Frankfurt am Main, Germany

**Keywords:** Peptic ulcer perforation, *Candida*, Antifungal therapy, Peritoneal swab, Gastric perforation, Duodenal perforation

## Abstract

**Purpose:**

The purpose of this study was to evaluate the outcome of patients with perforated peptic ulcer, stratified by detection of *Candida* spp. from peritoneal swabs.

**Methods:**

A retrospective, single-center, observational study was performed. All adult patients with perforated peptic ulcer who underwent surgical therapy were included. *Candida* spp. detection was defined as the result of culture incubation from peritoneal swabs at the index surgery. Its association with postoperative complications and in-hospital mortality was analyzed.

**Results:**

A total of 187 adult patients were included. Intraperitoneal pathogens were detected by microbiological analysis in 96 patients (61.9%). *Candida* spp. were detected in 62 patients (39.4%), of whom 23 (37.7%) received antifungal therapy. Patients with peritoneal detection of *Candida* spp. had an increased in-hospital mortality (OR 5.80, 95% CI 1.96–16.97, *p* < 0.001), and an increased rate of Clavien-Dindo grade III or higher complications (OR 6.08, 95% CI 2.95–12.54, *p* < 0.001), suture dehiscence (*p* = 0.003), and longer hospital stays (*p* = 0.002). The multivariable analysis revealed that severe complications (OR: 8.83; 95% CI: 3.19–24.46; *p* < 0.001) and in-hospital mortality (OR: 4.81; 95% CI: 1.16–19.90; *p* = 0.030) were significantly more prevalent among patients with peritoneal detection of *Candida* spp.

**Conclusion:**

Intraperitoneal presence of *Candida* spp. is associated with increased morbidity and mortality in patients with perforated peptic ulcer.

**Supplementary Information:**

The online version contains supplementary material available at 10.1186/s12893-026-03518-7.

## Introduction

Peptic ulcer results from acid-induced injury to the gastroduodenal mucosa. Dietary factors, stress, *Helicobacter pylori* infection, non-steroidal anti-inflammatory drugs, alcohol and tobacco abuse are common causes [1]. Perforated peptic ulcer is associated with a high level of morbidity and the mortality rate of up to 25% [2]. The incidence of positive fungal cultures obtained from intraoperative swabs in perforated peptic ulcers is reported to be in the range of 40% [2]. The impact of the detecting of *Candida* spp. in intraoperative swabs on patients´ outcome is controversially discussed. While some studies identified an association of the presence of *Candida* spp. at the surgical site with poorer outcomes [2–5], others have reported no significant difference [6] and no clear benefit from antifungal treatment [7–9]. The World Society of Emergency Surgery guidelines recommend antifungal therapy for patients at high risk for fungal infections, such as those with immunosuppression, advanced age, comorbidities, prolonged intensive care unit stays, or unresolved intra-abdominal infections [10].

The purpose of this study is to investigate the impact of intraoperative pathogen detection from intraoperatively taken swabs with a specific focus on *Candida* spp. on the clinical outcomes of patients suffering from perforated peptic ulcer disease, who were surgically treated.

## Materials and methods

This is a retrospective, single-center, observational study. It was approved by the local ethics committee of the Faculty of Medicine at the Goethe University Frankfurt, Germany (reference number: 2024 − 1695). All adult patients with perforated peptic ulcer, who underwent surgical therapy at the Department of General, Visceral, Transplant and Thoracic Surgery, University Hospital Frankfurt from January 2002 to February 2024, were included. Data were retrospectively collected from the electronic patients’ records. 32 patients (17.1%) who did not have an intraoperative swab culture were excluded from the analysis.

### Patients- and procedure-related parameters

Demographic parameters (age at surgery, sex), presence of pre-existing comorbidities, and current medications were recorded.

The location of the perforation was classified as follows based on surgical reports: gastric or duodenal. Other parameters recorded were postoperative complications according to the Clavien-Dindo classification (CDC) [11], postoperative suture dehiscence confirmed by the drainage fluid, computed tomography or reoperation, bleeding confirmed by endoscopy or reoperation on surgical site, laboratory parameters [C-reactive protein (mg/dL), white blood cells (/nL), creatinine (mg/dL) albumine (g/dL)], and histopathological findings with regard to malignancy and to *Helicobacter pylori*.

### Microbiological analysis

All laboratory tests were conducted under strict quality-controlled criteria (laboratory accreditation according to ISO 15189:2014 standards; certificate number D–ML–13102–01–00). All microbiological analyses were performed according to standard procedure as previously described [12]. In particular, *Candida* spp. were cultured by using Sabouraud agar (Oxoid, Wesel, Germany). Species identification (bacteria and *Candida* spp.) was done using the VITEK 2 system (bioMérieux, Nürtingen, Germany) and since 2010 by matrix-assisted laser desorption ionization-time of flight analysis (VITEK MS, bioMérieux). Antibiotic susceptibility testing was performed according to Clinical and Laboratory Standards Institute (CLSI) guidelines and since 2019 according to the recommendations of the European Committee on Antimicrobial susceptibility testing (EUCAST) using VITEK 2 and/or antibiotic gradient tests (bioMérieux) for bacteria and VITEK 2 (AST-YS 07/08) and/or Micronaut microdilution assay (Bruker Daltronics GmbH, Germany) for *Candida* spp [13]. All microbiological analyses were performed as previously described [12].

### Statistical analysis

Statistical analysis was performed using IBM SPSS version 29.0.0.2 software. Descriptive statistics with median and percentage of total for binary and categorical parameters as well as median and interquartile range (IQR) for continuous parameters were calculated.

Chi-squared tests were used to compare *Candida* spp. detected versus not detected from the intraoperative swab for the following parameters: in-hospital mortality, postoperative complications, severe complications (CDC ≥ III), need for reoperation, suture dehiscence, and bleeding. For comparison of length of hospital stay for discharged patients, the Mann-Whitney U test was applied.

Univariable binary logistic analysis was performed to identify the risk factors for in-hospital mortality and major postoperative complications in patients with perforated peptic ulcer. Age, a continuous variable, was converted into a variable indicating whether it was above or at the median. After identifying risk factors for outcome parameters (severe postoperative complications, CDC ≥ 3, and in-hospital mortality) multivariable analysis was performed to identify parameters with a significant effect on in-hospital mortality and major postoperative complications after adjusting age at median or above in years, male sex, presence of cardiovascular disease, previous malignancy history, use of anticoagulants, detection of *Candida* spp., laparoscopic surgery, surgical bleeding, suture dehiscence and *H. pylori*-eradication. Odds ratios (OR) and 95% confidence intervals (CI) were calculated.

## Results

### Baseline characteristics and surgical procedures

A total of 187 patients with perforated peptic ulcer were identified. Of these 116 (62%) were male and 71 (38%) were female. The median age at the time of surgery was 58 years (IQR 28).

Table 1 provides a detailed breakdown of the demographics and clinical characteristics of patients with perforated peptic ulcers.

**Table 1:** Demographic and clinical characteristics of patients with perforated peptic ulcer with regard to the detection of *Candida* spp

*N* = 187		Peritoneal swabs	Detection of Candida spp.		*p* value
N (%/IQR)		*N* = 155	*yes*	*no*	
Variables			*61(39.4)*	*94(60.6)*	
Age at the time of surgery [years]	58 (28)		65(29)	55(29)	< 0.011
Sex					0.311
Male	116 (62)	97 (62.6)	35(57.4)	62(64.9)	
Female	71 (38)	58 (37.4)	26(42.6)	32(34)	
Localization					0.857
Gastric	135 (72.2)	109 (70.3)	42(68.9)	67(71.3)	
Duodenal	52 (27.8)	46 (29.7)	19(30.6)	27(28.7)	
Surgical approach					0.806
Laparoscopic	27 (14.4)	19 (12.3)	8(13.1)	11(11.7)	
Open	160 (85.6)	136 (87.7)	53(86.9)	83(88.3)	
Antifungal therapy	35 (21.1)	34 (21.9)	23(37.7)	11(11.7)	< 0.001
Empirical *H. pylori* eradication	59 (21.6)				
Postoperative complications		73 (47.1)	40(65.6)	33(35.1)	< 0.001
CDC					
0	100 (53.5)				
I	10 (5.3)				
II	13 (7)				
IIIA	20 (10.7)	CDC ≥ 3	36(59)	18(19.1)	< 0.001
IIIB	13 (7)	54 (34.8)			
IV	6 (3.2)				
In-hospital mortality	25 (13.4)	20 (12.9)	15(24.6)	5(5.3)	< 0.001
Reoperation	24 (12.8)	19 (12.3)	8(13.1)	11(11.7)	0.086
Suture dehiscence	18 (9.6)	16 (10.3)	12(19.7)	4(4.3)	0.003
Surgical bleeding	7 (3.7)				
Length of hospital stay [days]	9(7)		11(12)	9(5)	0.002
Cardiovascular disease	68 (36.4)				
Previous malignancy history	25 (13.4)				
Chemotherapy	7 (3.7)				
Steroids	7 (3.7)				
Non-steroidal anti-inflammatory drugs	17 (9.1)				
Antiplatelet agents or anticoagulants	26 (13.9)				
Intervention up to 3 months prior to surgery	15 (8)				
Clinical signs of peritonitis	182 (97.3)				
Computed tomography	122 (65.2)				
Free air on computed tomography	120 (64.2)				
Free air on plain abdominal X-ray	49 (26.2)				
Intraoperative swab (*N* = 155)					
Intraoperative swab positive	96 (61.9)				
Intraoperative swab positive for *Candida*	61 (39.3)				
Perforation diameter					
<2 cm	136 (72.7)				
≥2 cm	26 (13.9)				
Procedure					
Primary suture	71 (38)				
Primary suture with omental patch	96 (51.3)				
Partial gastrectomy with reconstruction	17 (9.1)				
Bulbojejunostomy alone	2 (1.1)				
Roux-Y duodenojejunostomy	1 (0.5)				
T-drain	2 (1.1)				
Malignancy	0 of 162				
*H. pylori*-associated inflammation	10 (5.3)				
Follow-up [days]	25(730)				

*N* number, *IQR* interquartile range, *CDC* Clavien-Dindo classification

Intraoperative peritoneal swabs were performed in 155 patients (82.9%). No microorganisms were detected in 59 of these patients (38.1%). 61 patients (39.4%) had a detection of *Candida* spp. in the intraoperative peritoneal swabs. A detailed list of microorganisms identified in intraoperative peritoneal swabs is presented in the Supplementary Table 1.

### Outcome parameters after surgery for perforated peptic ulcer

Postoperative morbidity occurred in 87 patients (46,5%), including suture dehiscence in 18 patients (9.6%), surgical bleeding in seven patients (3.7%), and surgical site infection in 24 patients (12.8%). 64 patients (34.2%) had postoperative CDC ≥ 3 complications. The median length of hospital stay was nine days with an interquartile range of six days. The in-hospital mortality was observed in 25 patients (13.4%) [Table 1].

The postoperative morbidity was significantly higher in patients with *Candida* spp. detection (65.6%) compared to those without (35.1%, *p* < 0.001). In addition, CDC ≥ 3 complications were observed in 59% of patients with *Candida* spp. detection, compared to 19.1% in those without (*p* < 0.001). Patients with *Candida* spp. detection had significantly higher in-hospital mortality rates (15 patients [24.6%] versus five patients [5.3%] [*p* < 0.001]) and higher suture dehiscence rates (12 patients [19.7%] versus four patients [4.3%] [*p* = 0.003]). The length of hospital stay was longer for patients with *Candida* spp. detection (11 days; IQR 12 days) compared to those without (9 days; IQR 5 days, *p* = 0.002). There were no significant differences between patients with or without *Candida* spp. detection regarding gender, presence of cardiovascular disease or malignancy at the time of surgery, need for reoperation, location of the perforated peptic ulcer, or surgical approach laparoscopic versus open [Table 1].

Age ≥ 58 years, male sex, presence of cardiovascular disease, use of anticoagulants, detection of *Candida* spp. from peritoneal swabs, C-reactive protein, creatinine, albumin, postoperative surgical bleeding, postoperative suture, and need for reoperation were significantly associated with higher in-hospital mortality. In contrast diabetes mellitus, history of malignancy, use of antiplatelets, white blood cell count, gastric location of the perforation, performing a laparoscopic surgery, perforation diameter ≥ 2 cm, performing ulcer excision, antifungal therapy or empiric *H. pylori*- eradication did not show significant effects.

The following parameters had a significant effect on severe complications (CDC ≥ 3): age in years, presence of cardiovascular disease and malignancy, detection of *Candida* spp. from peritoneal swabs, C-reactive protein, creatinine, albumine, laparoscopic surgery, and empiric *H. pylori-*eradication.

Table 2 shows the results of univariable analysis in patients with perforated peptic ulcer with respect to in-hospital mortality and major postoperative complications.

**Table 2:** Univariable analysis of risk factors for postoperative mortality and major complications in patients operated for perforated peptic ulcer

	In-hospital mortality			Severe complications (CDC ≥ 3)		
	OR	95% CI	*p* value	OR	95% CI	*p* value
Age [≥ 58 years]	7.63	2.19–26.58	0.001	7.01	3.39–14.47	< 0.001
Male sex	0.38	0.16–0.92	0.032	0.57	0.31–1.05	0.072
Cardiovascular disease	3.51	1.32–9.31	0.012	2.77	1.45–5.27	0.002
Diabetes mellitus	1.30	0.26–6.48	0.749	0.75	0.21–2.63	0.653
History of malignancy	1.99	0.66–5.94	0.220	4.31	1.78–10.44	0.001
Anticoagulants	4.41	1.05–21.22	0.044	2	0.48–8.28	0.339
Antiplatelets	1.79	0.59–5.46	0.306	1.98	0.81–4.83	0.135
Detection of *Candida* spp.	5.80	1.96–16.97	0.001	6.08	2.95–12.54	< 0.001
C-reactive protein [mg/dL]	1.04	1.00-1.08	0.045	1.08	1.04–1.12	0.001
White blood cells [/nL]	0.97	0.90–1.04	0.414	0.97	0.92–1.02	0.257
Creatinine [mg/dL]	2.65	1.72–4.07	< 0.001	3.31	1.91–5.71	< 0.001
Albumine [g/dL]	0.18	0.07–0.48	< 0.001	0.33	0.17–0.61	< 0.001
Gastric localization	0.74	0.30–1.85	0.519	0.55	0.29–1.06	0.075
Laparoscopic surgery	0.23	0.03–1.77	0.158	0.13	0.03–0.55	0.006
Perforation diameter ≥ 2 cm	1.66	0.98–2.79	0.061	1.37	0.91–2.07	0.134
Ulcer excision	1.95	0.84–4.57	0.123	1.80	0.98–3.31	0.058
Surgical bleeding	10.68	2.23–51.14	0.003	N/A	N/A	N/A
Suture dehiscence	3.21	1.02–10.04	0.045	N/A	N/A	N/A
Reoperation	4.59	1.70–12.40	0.003	N/A	N/A	N/A
*Helicobacter pylori* eradication	0	0-N/A	0.997	0.341	0.17–0.70	0.003
Antifungal therapy	1.3	0.43–3.83	0.648	2.06	0.96–4.43	0.064

*CDC* Clavien-Dindo classification, *CI* confidence interval, *OR* Odds ratio

In multivariable analysis, only detection of *Candida* spp. from peritoneal swabs and postoperative surgical bleeding were found to be independent risk factors for in-hospital mortality. History of malignancy (OR: 9.55; 95% CI: 1.94–46.97; *p* = 0.005) and detection of *Candida* spp. from peritoneal swabs (OR: 8.83; 95% CI: 3.19–24.46; *p* < 0.001) were associated with higher rate of major postoperative complications, while performing laparoscopic surgery (OR: 0.08; 95% CI: 0.01–0.72; *p* = 0.025) was with a lower rate [Table 3].

**Table 3:** Multivariable analysis in patients with perforated peptic ulcer

	In-hospital mortality			Severe complications (CDC ≥ 3)		
	OR	95% CI	*p* value	OR	95% CI	*p* value
Age (≥ 58 years)	4.57	0.73–28.40	0.103	2.09	0.66–6.65	0.214
Male sex	0.92	0.14–1.78	0.279	-	-	-
Cardiovascular disease	1.41	0.33–6.05	0.642	1.26	0.41–3.91	0.683
Previous malignancy history	-	-	-	9.55	1.94–46.97	0.005
Anticoagulants	1.44	0.12–17.67	0.774	-	-	-
Detection of *Candida* spp.	4.81	1.16–19.90	0.030	8.83	3.19–24.46	< 0.001
Laparoscopic surgery	-	-	-	0.08	0.01–0.72	0.025
Surgical bleeding	11.47	1.61–81.93	0.015	-	-	-
Suture dehiscence	3.36	0.82–13.79	0.090	-	-	-
*H. pylori* eradication	-	-	-	0.45	0.18–1.12	0.086

*CDC* Clavien-Dindo classification, *CI* confidence interval, *OR* Odds ratio

## Discussion

The current study shows that patients with *Candida* detection during peptic ulcer surgery are at an increased risk for in-hospital mortality and have a higher rate of major complications, including suture dehiscence. These patients also have longer hospital stays. Additionally, the multivariable analysis revealed that severe complications and in-hospital mortality were significantly more prevalent among patients with peritoneal detection of *Candida* spp.

Other studies support an association between *Candida* infection and poor outcomes. Treuheit et al. showed higher 90-day mortality [2]. Shan et al. reported longer hospital stays, higher surgical site infection and mortality rates [3], and Li et al. showed higher incidence of bacteremia, extended intensive care unit and overall hospital stays, prolonged ventilation, and increased hospital mortality in patients with peritoneal candidiasis [5]. Conversely, in a study of 542 patients with a similar incidence of positive fungal isolates (38.6%), Kwan et al. found no effect on the outcome of patients with perforated peptic ulcer in multivariate analysis [6].

The use of empirical antifungal therapy remains a longstanding dilemma. A study date back to 1988 provide little to no evidence of benefit [14]. In the current study, only a minority of patients received antifungal therapy with no significant effect on outcomes. Despite the lack of evidence of fungal infection, 12 patients (6.4%) in the cohort received antifungal therapy. Antimicrobial stewardship interventions can significantly reduce the postoperative use of antimicrobial therapy for intra-abdominal infections [15]. Another earlier study suggested that early antifungal treatment for intra-abdominal candidiasis of gastrointestinal origin was independently associated with improved survival [16]. Controversially, Li et al. found no benefit of antifungal therapy, even in patients with positive cultures, but their number of patients was small and the duration of antifungal therapy was not standardized [9].

In our department, empiric antifungal therapy is initiated on a case-by-case basis considering patient’s clinical condition, comorbidities or immunosuppression, and laboratory parameters. While no benefit of antifungal therapy has been shown even in cases with detection of *Candida* peritonitis, it remains a dilemma both as an empiric and targeted treatment [9, 10, 14, 16]. The empirical administration of antifungal therapy is hindered by intrinsic *Candida* subtype resistance, side effects, resistance development, and costs. There are currently no randomized controlled studies on the benefits of the empiric therapy. The type, duration, and preparation of the antifungal therapy are considered to play a particularly important role. Several studies, including the current study, have demonstrated an association between detecting *Candida* spp. in peritoneal swabs and postoperative morbidity. However, the benefit of antifungal therapy remains unclear.

The multivariable analysis revealed that patients who underwent laparoscopic surgery experienced fewer severe postoperative complications. However, due to the small number of patients who underwent laparoscopic surgery, it is unclear why some patients were selected for laparoscopy instead of open surgery. Therefore, this result should be viewed critically. Although minimally invasive surgery is associated with fewer complications, it is possible that patients with fewer comorbidities were chosen for laparoscopy.

### Limitations

The retrospective nature of the study, the long 22-year patient cohort and non-standardized antifungal therapies are all limitations of the current study. Future prospective or multicenter studies with larger patient cohorts are needed to determine the exact association between *Candida* peritonitis and morbidity or mortality, as well as the necessity of antifungal therapy.

## Conclusion

Perforated peptic ulcer with a positive intraoperative *Candida* culture in peritoneal swabs is associated with increased postoperative morbidity.

## Supplementary Information

Supplementary Material 1.

## Data Availability

The datasets used and/or analyzed during the current study are available from the corresponding author upon reasonable request and after approval from Goethe University Digital Integration Center.
