# Supplementary material for: Detection of Candida spp. from peritoneal swabs indicate worse outcome in patients with perforated peptic ulcer: revisiting a longstanding debate
Source: BMC Surg. 2026 Jan 24;26:81. doi: 10.1186/s12893-026-03518-7 (PMC12849569; doi:10.1186/s12893-026-03518-7)
Supplement: Supplementary file 1 — Supplementary Material 1. [file 12893_2026_3518_MOESM1_ESM.docx]

| Supplementary Table 1 List of microorganisms in intraoperative peritoneal swabs, N=187 | |
| --- | --- |
|  | N/N(%) |
| No peritoneal swab | 32 (17.1) |
| **Peritoneal swab** | **155 (82.9)** |
| Sterile peritoneal swabs | 59 (38.1) |
| Positive peritoneal swabs | 96 (61.9) |
| **Positive Candida culture** | **61 (39.4)** |
| *Candida* spp. without subtyping | 18 |
| *C. albicans* | 16 |
| *C. glabrata* | 4 |
| *C. albicans, C. glabrata* | 1 |
| *C. albicans, C. krusei* | 1 |
| *C. albicans, C. glabrata, Streptococcus mitis/oralis, E. coli* and *Bacillus spp. eus* | 1 |
| *C. glabrata, P. mirabilis, E. coli* and *E. faecalis* | 1 |
| *C. albicans, C. tropicalis, E. faecalis, K. pneumoniae, E. coli* | 1 |
| *Candida* spp. *and Klebsiella* spp. | 3 |
| *C. glabrata* and *Klebsiella oxytoca* | 1 |
| *Candida* spp*. and Streptococcus* spp. | 4 |
| *C. krusei, C. albicans, S. anginosus* | 1 |
| *C. glabrata*, *E. coli* | 1 |
| *C. albicans, E. faecium* | 1 |
| *Candida* spp.*, E. coli* and *E. faecium,* | 1 |
| *C. krusei, E. coli* (extended spectrum beta-lactamase) | 1 |
| *Candida* spp*., E. coli* | 1 |
| *Candida* spp*., Klebsiella* spp*., Enterococcus* spp. | 1 |
| *Candida* spp., *Citrobacter* spp*.*, *Streptococcus* spp. | 1 |
| *Candida* spp., *Bacillus* spp. | 1 |
| *Candida* spp., *Lactobacillus fermentum* | 1 |
| *Streptococcus* spp. | 6 |
| *S.mitis/oralis* | 1 |
| *S. pyogenes* | 1 |
| *S. parasangiunis* | 2 |
| *S. parasanguis, S., salvarius* | 1 |
| *S. pneumoniae* | 1 |
| *Streptococci* (C-group) | 1 |
| *S. salivarius* | 1 |
| *S. pyogenes, Proteus mirabilis and Bacteroides species* | 1 |
| *S. mitis/oralis* and *Staphylococcus aureus* | 1 |
| *S. salivarius, R. mucilaginosa* | 1 |
| *Staphylococcus* spp. | 1 |
| *S. aureus* | 1 |
| *S. epidermidis* | 1 |
| *S. aureus, E. coli* | 1 |
| *E. faecalis* | 1 |
| *E. faecalis, M. luteus* | 1 |
| *E. faecium* | 1 |
| *E. faecium, Serratia* spp., *Stenotrophomonas* spp. | 1 |
| *E. coli* | 1 |
| *E. coli* (multidrug-resistant gram-negative bacteria) | 2 |
| *E. coli, Proteus* spp. | 1 |
| *E. coli*, *Bacteroides species* | 1 |
| *E. coli, E. faecium, E. aerogenes,* *S.cerevisiae* | 1 |
| *Pseudomonas* spp., *Serratia* spp., *Bacteroides* spp. | 1 |
| *Klebsiella* spp. | 1 |
| *V. atypica* | 1 |
| *C. acnes* | 1 |
| N- number |  |
